# Dental and dental hygienist trainee satisfaction with their veterans affairs clinical training experiences during the COVID-19 pandemic

**DOI:** 10.1186/s12909-024-05628-3

**Published:** 2024-06-11

**Authors:** Heather Northcraft, Jia Bai, Anne R. Griffin, Aram Dobalian

**Affiliations:** 1 Veterans Emergency Management Center (VEMEC), US, Department of Veterans Affairs, 16111 Plummer St. MS-152 Bldg. 22, North Hills, CA 91343 USA; 2 Division of Health Services Management and Policy, The Ohio State University College of Public Health, Columbus, OH USA

**Keywords:** Dental education, Career choice, COVID-19 pandemic, Dental hygienists

## Abstract

**Background:**

The COVID-19 pandemic rapidly altered dental practice, training, and education. This study investigates the pandemic’s impacts on the clinical training experiences of dental and dental hygienist trainees at the US Department of Veterans Affairs (VA).

**Methods:**

Using data from post-doctoral general practice dentists, dental specialists, and dental hygienist trainees who completed the VA Trainee Satisfaction Survey before and during COVID-19, we performed logistic regression and thematic content analyses to determine whether COVID-19 was associated with training satisfaction and likelihood of considering future VA employment.

**Results:**

While post-doctoral dentist and dental specialty trainees did not report significant differences, dental hygienist trainees reported increased overall satisfaction and an increased likelihood to consider future VA employment during the pandemic compared to before the pandemic. Similar reasons for dissatisfaction were identified for both the pre-pandemic and pandemic groups.

**Conclusions:**

Research outside VA indicates the pandemic’s association with trainees’ intentions to leave health profession education programs. Our results suggest the likely existence of factors that could lead to positive changes for at least some portion of the dental workforce. Future studies should explore those potential factors as some may be replicable in other settings or may apply to other health professions.

## Background

The COVID-19 pandemic rapidly altered dental practice and education in the United States (US) and around the world. For the safety of dental professionals and patients alike, the American Dental Association (ADA) issued guidance on March 16, 2020 that dental practices only provide emergency services or urgent care for patients [1]. According to a survey issued by the ADA, by March 23, 2020, 76% of dental practices were only seeing emergency patients while 19% of practices were closed and not seeing any patients [1]. By April 2020, employment in dental offices decreased to only 45% compared to January 2020 [1]. In addition to closures and unemployment during the pandemic, dentists experienced various declines in mental health [2, 3], increased job stress, and decreased job satisfaction [3, 4], all leading to heightened intentions to leave their chosen career [5]. Dental trainees, including predoctoral dental students, postdoctoral dental residents, and dental hygiene students, both in the US and around the world, also experienced similar adverse personal, educational, and professional outcomes due to the COVID-19 pandemic [6–14].

Research has continued to demonstrate the harmful effects of the COVID-19 pandemic on dental trainees’ personal and mental well-being (6–7, 11–13) across both differing dental educational levels (e.g., dental students and post-doctoral residents) and different dental programs (e.g., general practice, oral and maxillofacial surgery, dental hygiene). Dental trainees not only reported concerns regarding emotional health [12] but also suggested that their institutions should do more to address their mental well-being [11]. Nonetheless, less than 75% of programs offered options for stress reduction for residents during the pandemic (e.g., mindfulness seminars and yoga) [7]. Research has also found increased burnout following the COVID-19 pandemic [11]. Furthermore, those closer to graduation and those whose graduation plans changed as a result of the COVID-19 pandemic reported more anxiety compared to those without such circumstances [6].

In addition to the negative effects of the pandemic on their personal lives, trainees’ education and professional intentions were also disrupted in the early stages of the pandemic [7, 9, 10, 12–14]. Adverse educational outcomes included modified, decreased, and even suspended clinical experiences [7, 14]. Furthermore, studies have also reported that a majority of residents experienced negative impacts to their operative training [14], and many reported ineffective clinical experiences during their COVID-19 related school closures [12]. Subsequently, many trainees indicated that their educational or professional plans were altered due to the COVID-19 pandemic. Studies show that many dental trainees changed their future plans after the onset of COVID-19, with those closer to graduation indicating feeling more apprehensive about their future path due to concerns with meeting current accreditation graduation requirements and other factors [10, 14]. Dental residents and dental hygiene students indicated difficulties finding current or future employment and postponed or even discontinued future employment discussions during the pandemic [10, 14]. Some trainees were already considering different career plans as early as April 2020 [10].

One study with a mixed sample of dental students, graduate dental students and postdoctoral dental residents found that almost 13% reported intentions to leave their program [9]. In addition, the same study found that respondents who reported impacts of the pandemic on their mental health, which consisted of almost 70% of the sample, also reported an intention to leave their program [9]. Moreover, trainees expressed apprehension regarding the security of the dental profession as a whole [10]. Intention to leave one’s program and uncertainty in the safety of a career in dentistry is especially concerning when considering the future supply of oral health professionals. For example, research has shown decreased enrollment in dental hygienist programs after the pandemic [15]. Furthermore, there may be lingering reluctance to adequately staff dental health organizations following the pandemic. Data shows that across all types of dental healthproviders, staffing has been reduced and has yet to return to pre-COVID-19 pandemic numbers [16].

Like the rest of the world, dental care at the US Department of Veterans Affairs (VA) had to navigate challenges brought about by COVID-19, including adapting to new safety protocols and other technologies, in order to continue to deliver oral health services safely [17, 18]. While the VA previously had a history of strict infection control practices, VA dental professionals and trainees as a group still had to adapt to changes in practice necessitated by the COVID-19 pandemic [17, 18]. The VA patient population is more likely to be older and present with multiple comorbidities or other chronic conditions, including being immunocompromised, thus putting them at a higher risk for complications related to COVID-19 compared to non-veteran populations. To ensure the safety of VA patients as well as dental professionals and trainees alike, the VA instituted additional measures. These included plastic barriers, face shields, masks, the use of extraoral suction devices, and active coronavirus screenings [18]. Despite these measures, dental health profession trainees were still impacted by the pandemic as it interrupted training rotations during its initial stages [19].

The VA Office of Academic Affiliations (OAA) oversees all health professions education and training programs at VA facilities nationwide. The VA provides training for more than 118,000 healthcare professionals every year and sponsors approximately 33% of the US’s dental residency training programs [19, 20]. The VA has 70 dental residency programs nationally; in these programs, trainees receive clinical hands-on experiences and mentoring from dentists treating a Veteran population in both inpatient and outpatient settings. Almost half of dentists in postgraduate programs train in at least one rotation at a VA facility [21]. Therefore, understanding how dental and dental hygienist trainees at the VA were impacted by the pandemic is not only helpful to VA, but can also shed light on the pandemic’s overall impact nationwide.

Accordingly, researchers from the Veterans Emergency Management Evaluation Center (VEMEC) sought to investigate the impacts of the COVID-19 pandemic on the clinical training experiences of VA dental and dental hygienist trainees and to understand whether the pandemic may have been associated with changes in trainees’ attitudes toward future careers at the VA. Extant research regarding the impact of the COVID-19 pandemic on health professions’ trainees in US dental programs is limited in scope, limited to specific geographic regions, or has only included data obtained after the COVID-19 pandemic started. In contrast, our study uses a large, nationwide sample that includes data from both before and after the pandemic. Investigating the influence of disasters such as the COVID-19 pandemic on dental and dental hygiene trainees is important to understand its potential impacts on the size of the dental professional workforce and the nation’s oral health, especially in light of the shortages in dental professionals that existed before the pandemic but that were exacerbated during COVID-19 [15, 16].

## Methods

Every academic year, the OAA encourages all VA trainees to complete the Trainee Satisfaction Survey (TSS). The TSS is an opportunity for all clinical trainees to rate various aspects of their experience during their VA training, including the trainees’ satisfaction with their clinical training experience and consideration of future employment at a VA facility. The VA’s OAA then uses the feedback to identify areas of improvement. A majority of trainees complete a one-year rotation at a VA facility and at the conclusion of their rotations, all trainees are emailed a link to complete the TSS. Respondents answer the questions via an online survey at their own pace and are asked to complete the survey only once during the academic year. For the current study analyses, we used TSS data from three academic years which included responses collected from both before and during the COVID-19 pandemic (August 23, 2018 through July 21, 2021).

For this study, a dental trainee is defined as someone who completed one or more rotations at a VA facility and includes postdoctoral dental trainees in either general practice or in specialty areas (e.g., oral and maxillofacial surgery, prosthodontics), as well as dental hygienist trainees. Given the differences in training between general practice dentists, specialty dentists, and dental hygienists, the current sample was separated into two dental training groups: [1] dental trainees, which includes persons in postdoctoral general practice or a postdoctoral dental specialty and [2] dental hygienist trainees, which includes dental hygienist students with a minimum of an associate’s degree.

This study was approved as a quality improvement project by the Department of Veterans Affairs, Greater Los Angeles Institutional Review Board. All data used in the study are collected by OAA as part of its routine improvement efforts. Informed consent was waived by the Department of Veterans Affairs, Greater Los Angeles Institutional Review Board due to minimal risks to subjects. At the time of TSS data collection by OAA, respondents are assured of their anonymity. All data used in the study are collected by VA OAA as part of their routine improvement efforts, and participation in the survey is voluntary.

## Measures

Responses provided before February 29, 2020 were defined as “pre-pandemic”. Responses provided after April 1, 2020 were defined as “pandemic”. Responses during the month of March 2020 were not included in this study as it would be unclear from that data whether the respondent’s training period occurred either before or after the onset of the COVID-19 pandemic.

The TSS consists of 9 items related to levels of satisfaction with clinical training experience at the VA. If respondents indicated that they were either dissatisfied or very dissatisfied, they were subsequently asked to specify the cause of dissatisfaction in an open-ended text box. Those who indicated they were satisfied were not asked to provide additional information. Two TSS items were used to measure the main quantitative outcome variables of overall satisfaction (“Overall, how satisfied are you with your VA training experience?”) and likelihood of future employment at the VA (“As a result of your training experience, how likely would you be to consider a future employment opportunity at a VA medical facility?”).

### Statistical analyses

We used logistic regression to test the relationship of the pandemic group, dental training group (1 and 2; see above), and a pandemic group-by-dental training group interaction term for the outcome variable of overall satisfaction. Margin commands were subsequently used to calculate the difference in the probability of reporting either satisfied or dissatisfied for each group against p-value < 0.05. Margin commands were also used to calculate the 95% confidence interval for each group difference. The same analysis was completed for the outcome variable of likelihood of future VA employment. STATA SE version 17 was used to perform the analyses.

### Thematic content analysis

The qualitative data in this study were derived from narrative survey feedback provided by VA dental and dental hygienist trainees to the open-ended dissatisfaction responses in the TSS. As the TSS is a web-based, written survey, the narrative written responses were typically brief (range: 1–30 words with a median word count of 14). We employed a thematic content analysis to examine the open-ended narrative responses [22, 23]. All four members of the research team conducted the qualitative analyses. J.B. created initial themes, codes, and corresponding definitions based on the survey questions. All four members of the research team as a group conducted a review to identify any further issues in addition to the survey questions. J.B. and H.N. then conducted independent coding to assign each narrative a code(s). The entire research team met to discuss discrepancies between J.B.’s and H.N.’s original coding. Finally, frequencies were calculated for each dissatisfaction theme for both the pre-pandemic and pandemic periods. For additional information regarding qualitative analyses please see Northcraft et al. 2022 [24].

## Results

**Fig. 1:**
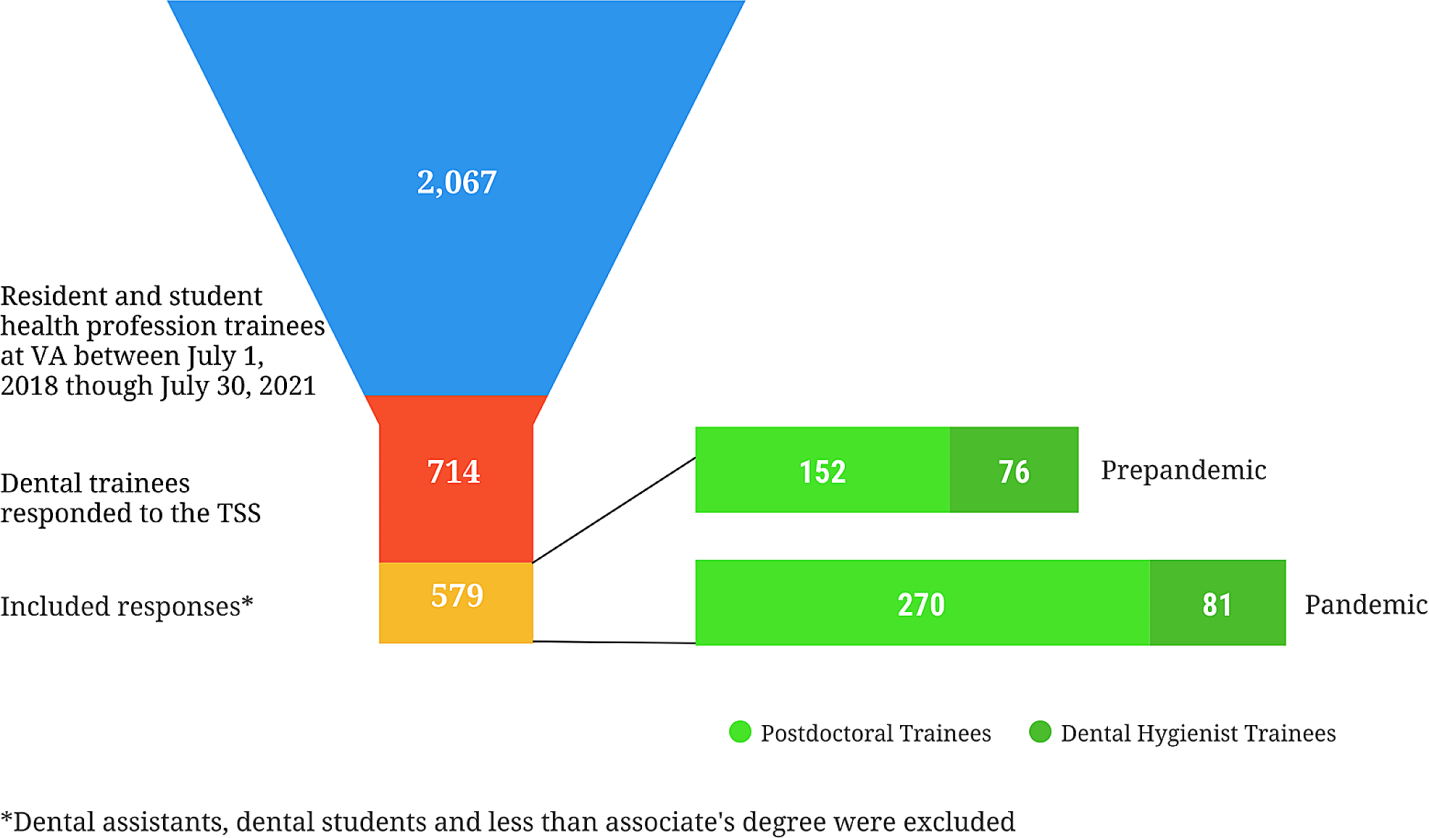
Final sample groups

A total of 2,607 dental residents and students received training in the form of at least one rotation at a VA health care facility in academic years 2018–2019 (July 1, 2018 – June 30, 2019), 2019–2020 (July 1, 2019 – June 30, 2020), 2020–2021 (July 1, 2020 – June 30, 2021) [19]. Across the three academic years, 714 trainees in the dental program responded to the TSS, yielding a 27.39% response rate. The current analyses consisted of 579 respondents, including only postdoctoral general practice dentists and postdoctoral dental specialties trainees (*n* = 422) as well as dental hygienist trainees with a minimum of an associate’s degree (*n* = 157) (see Fig. 1).

### Overall satisfaction and likelihood of future employment

While the pandemic group by dental training group interaction was not significant, there were notable differences between dental training groups (i.e. the postdoctoral trainees in Group 1 and the dental hygienist trainees in Group 2) (see Table 1). Although Group 1 did not report any significant differences for either overall satisfaction or likelihood of future VA employment, Group 2 reported significant increases for both outcome variables after the pandemic began. Dental hygienist trainees during the pandemic group reported increased overall satisfaction compared to their pre-pandemic counterparts (98.8% v. 90.8%; *p* = .0241). Correspondingly, dental hygienist trainees in the pandemic group also reported an increased likelihood to consider future VA employment (80.3% v. 60.5%; *p* = .0058).

**Table 1:** Logistic regressions predicting overall satisfaction and likelihood of future VA employment

	**% Trainees satisfied** **(95% CI)**, ***p***	**% Trainees likely** **(95% CI)**, ***p***	
**Group 1**:Post-doctoral General Practice Dentist and Specialist Trainees			
Pre-pandemic	89.5	56.6	
Pandemic	90.0	63.7	
	(-5.52%, 6.58%), *p* > .05	(-2.62%, 16.87%), *p* > .05	
**Group 2**:Dental Hygienist Trainees			
Pre-pandemic	90.8	60.5	
Pandemic	98.8	80.3	
	(1.04%, 14.91%), *p* = .0241	(5.72%, 33.72%), *p* = .0058	

### Dissatisfaction themes

Of the 579 dental trainee respondents, 178 provided narrative comments (approximately 31%). Seventy-one respondents provided comments during the pre-pandemic period (about 12%) and 107 did so during the pandemic period (approximately 18%). A review of dental and dental hygienist trainees’ narrative comments identified eight themes (see Table 2). Generally, the ranked frequency of each dissatisfaction theme was similar for both the pre-pandemic and pandemic groups. The most common cause of dissatisfaction both before and during the pandemic was the VA’s onboarding process, which includes receiving a VA identification badge and access to VA information systems. In general, trainees were dissatisfied with the delays and difficulties in the process to receive the VA badge. For example, trainees often reported not only several trips in order to obtain an ID badge but also an overall lack of communication and organization regarding the onboarding process as a whole. Another theme that consistently arose for both the pre-pandemic and pandemic groups was dissatisfaction with the working environment. For example, a number of respondents specifically mentioned that they perceived little sense of teamwork during training and many responses indicated perceptions of feeling unwelcomed as a trainee. In addition, trainees specifically felt that, as new trainees, they were treated as a nuisance by the established staff. Again, for both the pre-pandemic and pandemic groups, another common dissatisfaction theme was a lack of access to adequate workspaces or necessary procedure equipment or instruments. For example, respondents often mentioned slow or outdated computers with difficult to use software, which they reported hampered their learning opportunities and overall productivity and workflow. Trainees also communicated issues with access to necessary equipment such as working dental chairs or x-ray machines. In addition to these three areas of dissatisfaction, other areas included difficulties with their clinical trainer(s), issues with safety guidelines, and concerns regarding the cleanliness of the facilities. As mentioned, the dissatisfaction themes for both before and during the pandemic were similar, however two dissatisfaction themes were more present during the pandemic; these two themes included the volume and case mix of patients as well as issues with their rotations. For example, after the pandemic began, respondents mentioned a decrease or even a complete halt of some dental procedures due to the pandemic, as well as the low number of patients overall due to restrictions on various dental procedures during COVID-19.

**Table 2:** Narrative responses for trainee dissatisfaction pre-pandemic and during the pandemic

Theme	Pre-pandemic (*n* = 71)	%	Pandemic (*n* = 107)	%
Onboarding process (e.g., delays in VA badge and access)	46	64.8%	67	62.6%
Work environment (e.g., unwelcoming, no teamwork, hostile, no support staff)	30	42.3%	35	32.7%
Access to adequate workspace, functional computers, dentist chairs, x-ray machines, other instruments	28	39.4%	31	29.0%
Clinical trainer (e.g., faculty, hygienist)	15	21.1%	20	18.7%
Safety guidelines and cleanliness	11	15.5%	9	8.4%
Patient (volume, case mix)	2	2.8%	11	10.3%
Access to supplies	2	2.8%	4	3.7%
Rotation stopped	1	1.4%	4	3.7%

## Discussion

Postdoctoral general practice dentists and dental specialty trainees did not report any significant differences for either outcome variable; however, dental hygienist trainees during the pandemic reported increased overall satisfaction and an increased consideration for future employment at a VA facility compared to the pre-pandemic dental hygienist group. This result contrasts with previous research among both practitioners and trainees as a whole, who found negative experiences when COVID-19 impacted their profession and training. For example, dentists reported decreased job satisfaction and intentions to leave their career [4, 5]. Similar results were found among non-VA samples of various dental trainees including predoctoral students, postdoctoral residents (e.g., oral and maxillofacial surgery residents) as well as dental hygienist trainees who all reported generally negative experiences during the pandemic. Previous studies have shown that not only was training interrupted and adversely affected by the pandemic [7, 9, 10, 12, 14], but dental trainees’ future plans in the dental profession had changed [10, 14]. Some trainees reported intentions of leaving their training programs altogether as they expressed concerns about the dental profession as a whole including long-term impacts of closures and reduced volume of patients [9, 10]. Additionally, this result differs from a previous study among physician trainees at the VA which found that the pandemic was associated with both outcomes decreased satisfaction with training experience and decreased likelihood to consider future employment at a VA facility [24].

Given that the narrative data corresponding to dissatisfaction was also similar for both the pre-pandemic and pandemic groups, it is perhaps unsurprising that the COVID-19 pandemic was not correlated with more dissatisfaction among dental and dental hygienist trainees. However, the static satisfaction levels among dental trainees and increased satisfaction among dental hygienist trainees, combined with increased consideration of future VA employment among dental hygienist trainees after the onset of the pandemic, suggests the possibility of factors that made VA employment both [1] relatively more attractive for dental hygienists than other practice settings and [2] that the overall training environment was comparable to or better than other available practice settings in ways that the trainees felt were important. Because of the limitation inherent in the collected data, the present study cannot provide insights into what those factors may be, but there are possible considerations that should be explored in future studies to determine whether these factors are indeed influential and whether they are applicable in other settings and mutable. For example, post-doctoral residents have invested a significant amount of time in their education and training, and although the COVID-19 pandemic created challenges, the drive to continue in their chosen profession, at least with the VA, outweighed the obstacles presented by the pandemic. Another possible factor could be the advantages of working for a large employer, such as the VA. For instance, 53% of dental practices do not provide health insurance to their employees, [25] which would typically include dental hygienists, whereas the VA provides health insurance as well as other employee benefits. Additionally, as the VA healthcare system typically provides a unified, single healthcare facility in a given community, Veteran patients could be readily screened for COVID-19 onsite without the patient encumbering out-of-pocket expenses, and once the onsite VA laboratory reported negative results, the patient could easily be appointed for dental care. In contrast, dental practices in the private sector would typically need to request that their patients undergo a COVID-19 test performed by a private medical laboratory, which would add additional costs to dental procedures and increase the burden on the practice by necessitating that the practice obtain the patient’s results under the Health Insurance Portability and Accountability Act’s restrictions (because of concern about receiving unverified results directly from patients themselves). Accordingly, future studies should explore whether this finding is evident in other integrated health systems where dental services are available onsite. In addition, the cost of personal protective equipment (PPE) to protect the dentists, dental hygienists, and other staff is a cost that is not easily transferred to private dental patients, while that cost is not a factor within the VA at the level of the dental practitioner. It is also possible that PPE may have been more readily available within VA than within private dental practices because of differences in supply constraints, particularly within the first year after the pandemic started. As evidence to support this possibility, it should be noted that the ADA wrote letters to the Federal Emergency Management Agency and the U.S. Department of Health and Human Services expressing concern about the “cost, availability, and distribution of personal protective equipment” in December 2020 [26].

The current study has limitations. While respondents did complete the TSS throughout the academic year and OAA believes that the majority of respondent trainees that complete the survey do so relatively near the time that they completed their VA rotation, we could not identify the exact dates of training for the respondents. Additionally, TSS response rates across all VA trainee groups are historically low (11–14%) and therefore the collected data may not be representative of the perspectives and views of trainees who chose not to respond. However, the responses of the dental and dental hygienist trainees spanned 92 VA facilities spread broadly across the US, and we note that the response rate for the dental trainee group was higher than other healthcare profession groups. Finally, the open-ended narrative feedback was only solicited if the respondent indicated a dissatisfied response. Thus, potential positive outcomes arising from the pandemic could not be investigated. Solicitation of positive feedback may help future studies determine possible mitigating or protective factors the VA afforded to dental trainees.

## Conclusions

While it seems that the dental workforce has not yet fully recovered from the pandemic [15, 16] and although dental trainees have generally indicated that COVID-19 was a factor in their intention to leave their training programs or change career plans [9, 10], the current data suggest that there may be factors that could mitigate some of these negative effects. This is evident from our finding that VA dental and dental hygienist trainees likelihood to consider future VA employment was either unchanged or actually increased after the pandemic began. Future investigations should attempt to ascertain what those factors are that led to the reported increased desire to work for VA as some or all of these factors may be replicable in other settings and could lead to positive, long-term changes for the dental and dental hygienist workforces overall, and potentially other health professions that also face also workforce shortages.

## Data Availability

The Office of Academic Affiliations, US Department of Veteran Affairs granted data access to the research team through a process that is open to all researchers.
